# Real-World Data on Newly Diagnosed *BRCA*-Mutated High-Grade Epithelial Ovarian Cancers: The French National Multicenter ESME Database

**DOI:** 10.3390/cancers14164040

**Published:** 2022-08-21

**Authors:** Marta Bini, Stanislas Quesada, Pierre Meeus, Manuel Rodrigues, Eric Leblanc, Anne Floquet, Patricia Pautier, Frédéric Marchal, Magali Provansal, Loïc Campion, Sylvain Causeret, Sophie Gourgou, Isabelle Ray-Coquard, Jean-Marc Classe, Christophe Pomel, Thibault De La Motte Rouge, Emmanuel Barranger, Aude Marie Savoye, Cécile Guillemet, Laurence Gladieff, Martin Demarchi, Roman Rouzier, C Courtinard, Clémence Romeo, Florence Joly

**Affiliations:** 1 Centre Léon Bérard, 69008 Lyon, France; 2 Instituto Nazionale dei Tumori, 20133 Milano, Italy; 3 Institut Régional du Cancer de Montpellier, 34298 Montpellier, France; 4 Institut Curie, 75005 Paris, France; 5 Centre Oscar Lambret, 59000 Lille, France; 6 Institut Bergonié, 33076 Bordeaux, France; 7 Gustave Roussy, 94805 Villejuif, France; 8 Institut de Cancérologie de Lorraine, 54519 Vandœuvre-lès-Nancy, France; 9 Institut Paoli Calmettes, 13009 Marseille, France; 10 Institut de Cancérologie de l’Ouest, 44805 Saint-Herblain, France; 11 Centre Georges Francois Leclerc, 21000 Dijon, France; 12 Department of Surgery, Institut de Cancerologie de l’Ouest, Boulevard Professor Monod, 44805 Saint Herblain, France; 13 Centre Jean Perrin, 63011 Clermont-Ferrand, France; 14 Centre Eugene Marquis, 35000 Rennes, France; 15 Centre Antoine Lacassagne, 06100 Nice, France; 16 Institut Godinot, 51726 Reims, France; 17 Centre Henri Becquerel, 76038 Rouen, France; 18 Institut Claudius Regaud, IUCT Oncopole, 31059 Toulouse, France; 19 Institut de Cancérologie Strasbourg ICANS, 67200 Strasbourg, France; 20 Centre François Baclesse, 14000 Caen, France; 21 Unicancer, 75654 Paris, France

**Keywords:** *BRCA1*, *BRCA2*, high-grade epithelial ovarian cancer, real-world data, epidemiology, overall survival, platinum-based chemotherapy, progression-free survival, treatment patterns, epidemiological strategy and medical economics database

## Abstract

**Simple Summary:**

*BRCA*-mutated high-grade epithelial ovarian cancers represent a specific subset of gynecological malignancies. Real-world comprehensive data have been elusive to date. As such, we conducted a comprehensive description of clinicopathological and therapeutical characteristics via the Epidemiological Strategy and Medical Economics (ESME) data warehouse, which collects data from 18 French comprehensive cancer centers from the Unicancer network. This led to useful findings regarding the natural disease history of these patients in clinical practice, prior to the advent of poly-ADP ribose polymerase inhibitors.

**Abstract:**

Background: In spite of the frequency and clinical impact of *BRCA1/2* alterations in high-grade epithelial ovarian cancer (HGEOC), real-world information based on robust data warehouse has been scarce to date. Methods: Consecutive patients with *BRCA*-mutated HGEOC treated between 2011 and 2016 within French comprehensive cancer centers from the Unicancer network were extracted from the ESME database. The main objective of the study was the assessment of clinicopathological and treatments parameters. Results: Out of the 8021 patients included in the ESME database, 266 patients matching the selection criteria were included. *BRCA1* mutation was found in 191 (71.8%) patients, while 75 (28.2%) had a *BRCA2* mutation only; 95.5% of patients received a cytoreductive surgery. All patients received a taxane/platinum-based chemotherapy (median = six cycles). Complete and partial response were obtained in 53.3% and 20.4% of the cases, respectively. Maintenance therapy was administered in 55.3% of the cases, bevacizumab being the most common agent. After a median follow up of 51.7 months, a median progression-free survival of 28.6 months (95% confidence interval (CI) [26.5; 32.7]) and an estimated 5-year median overall survival of 69.2% (95% CI [61.6; 70.3]) were reported. Notably, *BRCA1*- and *BRCA2*-mutated cases exhibited a trend towards different median progression-free survivals, with 28.0 (95% CI [24.4; 32.3]) and 33.3 months (95% CI [26.7; 46.1]), respectively (*p*-value = 0.053). Furthermore, five-year OS for *BRCA1*-mutated patients was 64.5% (95% CI [59.7; 69.2]), while it was 82.5% (95% CI [76.6; 88.5]) for *BRCA2*-mutated ones (*p*-value = 0.029). Conclusions: This study reports the largest French multicenter cohort of *BRCA*-mutated HGEOCs based on robust data from the ESME, exhibiting relevant real-world data regarding this specific population.

## 1. Introduction

There were 313,959 new cases and 207,252 deaths reported in 2020 due to epithelial ovarian cancers (which includes ovarian, fallopian tube, and primitive peritoneal cancers), making this group of cancers the 8th most-incident and lethal cancers in women [1]. Furthermore, they represent the leading cause of death among cancers of the female reproductive system. This high lethality is partly explained by the absence of effective population screening strategies and by the lack of symptoms in early stages of the disease. Indeed, 75–80% of ovarian cancers are diagnosed at advanced stages (i.e., stages III and IV from the International Federation of Gynecology and Obstetrics or FIGO). This delayed diagnosis at an advanced stage has a major prognostic impact: while the 5-year overall survival (OS) for all stages combined is around 40%, there is a significant variation between stages I-II and stages III-IV (80–95% versus 10–30%, respectively) [2].

At the pathological scale, epithelial ovarian cancers are mainly represented by high-grade epithelial ovarian cancers (HGEOC), which represent 70% of the cases [3]. Molecularly, around 50% of HGEOC are characterized by homologous recombination deficiency (HRD), leading to subsequent genomic instability and higher sensitivity to platinum salts and poly-ADP ribose polymerase inhibitors (PARPi) [4,5]. Alterations in *BRCA1* and *BRCA2* genes are the most frequent and best-characterized causes of HRD so far. Indeed, 15% of patients with HGEOC carry a germline mutation in one of these genes, leading to an increased risk of developing HGEOC, with a cumulative risk of approximately 44% and 17% (*BRCA1* and *BRCA2*, respectively), versus 1.4% in the general population [4,6]. Apart from germline mutations, somatic mutations in *BRCA1* or *BRCA2* are found in an additional 4% and 3% of cases, respectively [7]. *BRCA*-mutated tumors have been reported to exhibit specific features, such as enhanced sensitivity to antineoplastic agents [8]. Nevertheless, *BRCA1* and *BRCA2* may have distinct impact on prognosis; several studies suggested that *BRCA2* mutations are associated with a better prognosis than *BRCA1*-mutated and non-mutated patients [9,10]. As such, *BRCA*-mutated HGEOC represent a specific subset of patients that needs to be specifically assessed.

For advanced HGEOC, the gold standard for upfront treatment remains debulking cytoreductive surgery with an objective of maximal cytoreduction with complete resection, usually associated with platinum- and taxane-based chemotherapy, which can be neoadjuvant or adjuvant [11,12]. Seventy percent of patients have an initial response to platinum salts; still, relapse is observed in roughly 75% of cases [2]. As such, novel targeted therapies have emerged in order to improve HGEOC prognosis. Based on several randomized clinical trials, bevacizumab (a monoclonal antibody targeting the vascular endothelial growth factor) has been shown to be effective on progression-free survival (PFS) when associated with systemic chemotherapy as first-line maintenance treatment [13,14,15]. More recently, the advent of PARPi (as maintenance, either in monotherapy or in combination with bevacizumab) led to a breakthrough in HGEOC management, with the most impressive responses seen in *BRCA*-mutated and HRD populations [16,17,18,19].

Apart from the impressive results of these data, real-life clinical practice specifically needs to be assessed in order to evaluate the true impact of therapeutic strategies. As such, real-world data analyses have been proposed as a way to bridge the gap between clinical trials and real-life clinical practice. Meanwhile, studies that evaluate real-world data frequently suffer from lack of standardization during data collection process, leading to potential bias. In this perspective, the Epidemiological Strategy and Medical Economics (ESME) research program has been developed, based on data collected from French Comprehensive Cancer Centers, with the aim to provide independent and high-quality real-world data [20]. Several studies have already been published so far, mainly focused on the ESME metastatic breast cancer database, with over 22,000 patients included [21,22,23,24,25,26,27,28,29,30]. Apart from metastatic breast cancer, another data platform has been developed, focusing on ovarian cancers. This data platform is an electronic health records-derived database populating data on consecutive patients diagnosed and/or treated for ovarian cancers (all-stage included) between 2011 and 2017 in one of the 18 French Comprehensive Cancer Centers. It offers a large real-world multicenter cohort, with demographics, tumor characteristics, clinical features, clinical events, and treatment-related data that may lead to a better knowledge of ovarian cancer disease history. Based on the ESME ovarian cancer platform, two studies that assessed the specificities of endometrioid ovarian cancers and the optimal timing of debulking surgery have been published recently [31,32].

Owing to the importance of *BRCA1* and *BRCA2* alterations, the present study specifically explored *BRCA*-mutated advanced HGEOC, with the aim to describe clinicopathological characteristics and treatment patterns in a real-world cohort.

## 2. Materials and Methods

### 2.1. Study Design and Selection Criteria

The ESME Ovarian Cancer (ESME-OC) database is a real-life French retrospective multicenter database centralizing clinical data of all consecutive patients treated for an ovarian cancer since 1 January 2011 at one of the 18 French Comprehensive Cancer Centers from the Unicancer network. In compliance with French regulations, the ESME OC database (NCT03275298) was authorized by the French data protection authority (Registration ID 1976564 and authorization N°DE-2017-311). Moreover, in compliance with the applicable European regulations, a complementary authorization was obtained on 14 October 2019 regarding the ESME research data warehouse. No formal dedicated informed consent was required, but participating centers handle processes to ensure that patients are informed about the re-use of their electronically recorded data. The ESME OC database centralized prospectively collected data from electronic medical records, inpatient hospitalization records, and pharmacy records. The full methodology has been previously described [31]. Data extraction date was 9 January 2019. Selection criteria were as follows: diagnosis of de novo advanced high-grade epithelial ovarian cancer (serous and/or endometrioid), age ≥ 18 years at diagnosis, *BRCA*-mutated (*BRCA1* and/or *BRCA2*), initial treatment occurring between 2011–2016 with platinum-based chemotherapy (+/− surgery) in first-line, absence of progression within 8 weeks after the end of first-line platinum-based chemotherapy. Data concerning age, Eastern Cooperative Oncology Group Performance Status (PS), CA-125 level, FIGO stage, histological type, locoregional invasion and metastatic sites at diagnosis, familial history of breast/ovarian cancer, personal history of cancer, *BRCA* mutation status, date of first and second disease progression, number of treatment lines, characteristics and timing of surgery, number of cycles of platinum-based chemotherapy as first-line treatment, type of chemotherapy, best response to first-line and second-line treatments, presence of maintenance treatment, date of first/second progression, and last date of follow up of were collected.

### 2.2. Primary and Secondary Objectives

The primary objective of the present study was a description of patient’s characteristics, clinical features, and treatment patterns of selected population from the ESME-OC database. Secondary objectives were assessment of survival criteria.

### 2.3. Statistical Analyses

For qualitative variables, baseline characteristics were summarized using frequency and percentage. Median and inter-quartile range, as well as mean and standard deviation, were reported for quantitative variables. Progression-free survival (PFS) was defined as the time from beginning of systemic first-line treatment for metastatic disease until the earliest documented disease progression or death for any cause in case of absence of progression or end of follow-up or the cutoff date. Progression-free survival 2 (PFS2) was defined as the time from beginning of systemic first-line treatment until the second-earliest documented disease progression or death for any cause in case of absence of progression or end of follow-up or the cutoff date. Time to first subsequent therapy (TFST) was defined as the time from beginning of first-line treatment until first subsequent therapy or death. Overall survival (OS) was defined as the time from beginning of systemic first-line treatment to the date of death (for any cause) or end of follow-up or cutoff date. PFS, PFS2, TFST, and OS were given as median values (mPFS, mPFS2, mTFST, and mOS, respectively). All median survival criteria were estimated using the Kaplan–Meier method, and median survival times were reported with their respective 95% confidence interval (95% CI). These analyses were subsequently stratified according to *BRCA* mutation status, FIGO staging, and presence of maintenance treatment. For PFS and OS, the survival distribution between subgroups was compared using the Log-rank test. Data were analyzed using SAS software (v9.2).

## 3. Results

### 3.1. Clinicopathological Features

**Figure A1:**
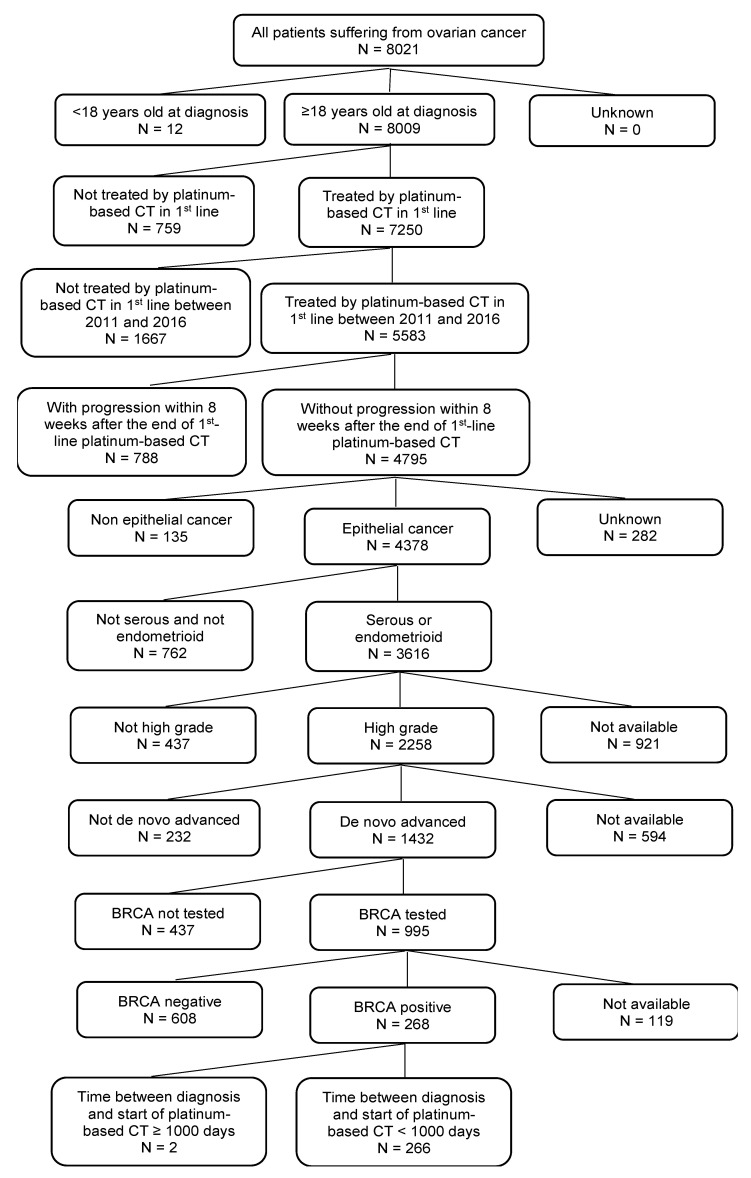
Flowchart.

Out of the 8021 patients included in the ESME-OC database, 266 patients matching the selection criteria were included (flowchart is provided in Figure A1). Detailed demographics are listed in Table 1.

Briefly, median age at diagnosis was 56.8 (range 33–81; standard deviation +/− 10.2). For 79 (29.6%) patients, PS was available upon diagnosis; the vast majority of these exhibited a PS of 0 (*n* = 107; 40.5%) or 1 (*n* = 125; 46.8%), with only a minority with a higher one (PS2: *n* = 24 or 8.9%; PS3: *n* = 10 or 3.8%). Baseline CA-125 was reported for 167 (62.7%) patients and a high value (i.e., >35 U/mL) was present in 161 (96.4%) cases. *BRCA1* mutation was found in 187 (70.3%) patients, while 75 (28.2%) had a *BRCA2* mutation; two (0.8%) patients carried mutations in both genes, and for two (0.8%) patients, the gene affected (i.e., *BRCA1* and/or *BRCA2*) was not specified. Notably, a familial history of breast or ovarian cancer was reported in 95 (49.7%) and 24 (32%) patients (*BRCA1* and *BRCA2,* respectively); conversely, personal history of previous cancer was reported in only three (1.1%) patients. Regarding disease, FIGO stage III was the most frequent stage with 129 (66.9%) patients, as for the serous-only carcinoma histological subtype (*n* = 185 or 96.6%). At diagnosis, hepatic, pulmonary and peripheral nodes metastasis were observed in 52 (19.5%), 13 (4.9%), and 100 (37.8%) patients, respectively. Interestingly, *BRCA1*-mutated and *BRCA2*-mutated seemed to present different trends of metastases (liver: 23.7% vs. 8.7%; lung: 1.7% vs. 13.0%; peripheral nodes: 35.6% vs. 43.5%, for *BRCA1*-mutated and *BRCA2*-mutated, respectively).

### 3.2. Treatment Characteristics

Detailed treatment patterns are specified in Table 2. Two hundred fifty-four (95.5%) patients received a cytoreductive surgery. Twenty-seven (38.0%) *BRCA2*-mutated patients and 87 (47.5%) *BRCA1*-mutated patients had primary debulking surgery. All patients received a taxane added with the platinum-based chemotherapy, with a median number of six cycles. Complete and partial response were obtained in 81 (53.3%) and 31 (20.4%) cases, respectively. Maintenance therapy was administered in 147 (55.3%) patients, with bevacizumab in the vast majority (78.9% as monotherapy and 12.9% in combination).

Most patients received only one line of treatment; nevertheless, 58 (21.8%) patients received at least four lines of systemic therapy. Regarding the second-line setting, an objective response rate (i.e., complete/partial response or stable disease) was observed in 71 (68.2%) patients (51 (69.3%) and 19 (65.5%) for *BRCA1* and *BRCA2*, respectively).

### 3.3. Survival Analyses

**Figure 1:**
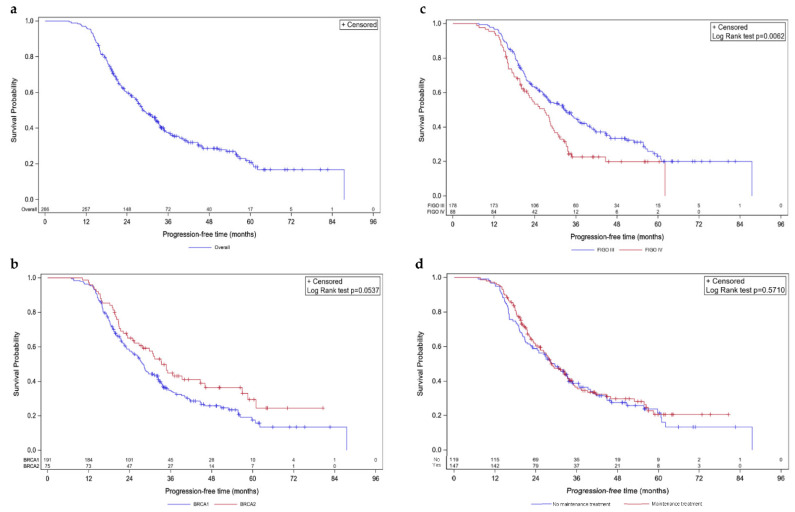
Kaplan–Meier progression-free survivals. Survival curves are given for overall population (**a**) and stratified according to mutated *BRCA1* vs. *BRCA2* (**b**), FIGO III vs. IV (**c**), and maintenance vs. no maintenance treatment (**d**).

**Figure 2:**
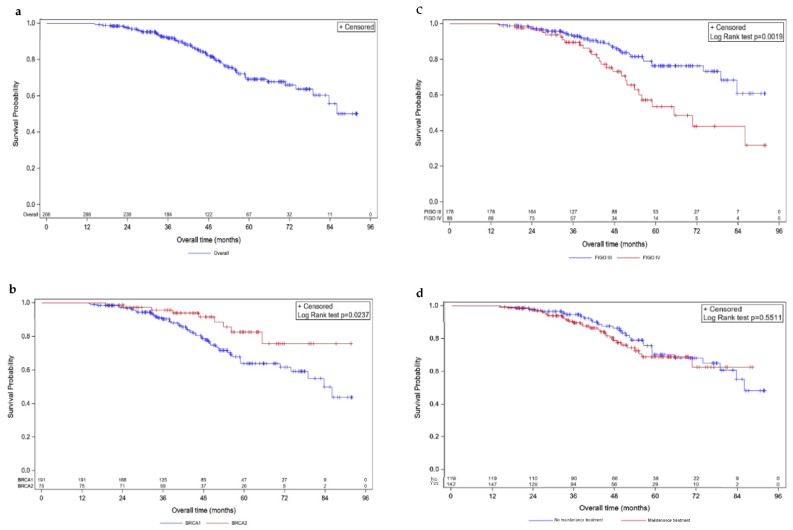
Kaplan–Meier overall survivals. Survival curves are given for overall population (**a**) and stratified according to mutated *BRCA1* vs. *BRCA2* (**b**), FIGO III vs. IV (**c**), and maintenance vs. no maintenance treatment (**d**).

After a median follow up of 51.7 months, distinct trends were observed regarding both PFS (Figure 1) and OS (Figure 2).

In the overall population, mPFS was 28.6 months (95% CI [26.5; 32.7]; Figure 1a). Concerning molecular status, a trend towards statistical difference was observed between *BRCA1*- and *BRCA2*-mutated cases, with an mPFS of 28.0 (95% CI [24.4; 32.3]) and 33.3 months (95% CI [26.7; 46.1]), respectively (*p*-value = 0.0537; Figure 1b). Patients with FIGO stage III and stage IV diseases had different mPFS with 32.7 (95% CI [27.1; 37.4]) and 26.7 months (95% CI [20.8; 29]), respectively (*p* = 0.0062; Figure 1c). Noteworthy, no difference was observed regarding the presence of maintenance treatment; patients that received one had an mPFS of 28.5 months (95% CI [25.4; 33.3]) versus 29.5 months (95% CI [23.1; 33.6]) for patients that did not (*p*-value = 0.5710; Figure 1d).

Median OS was not reached at the time of data extraction. The estimated 5-year mOS was 69.2% (95% CI [61.6; 70.3]; Figure 2a). Five-year OS for *BRCA1*-mutated patients was 64.5% (95% CI [59.7; 69.2]), while it was 82.5% (95% CI [76.6; 88.5]) for *BRCA2*-mutated ones, revealing distinct OS patterns (*p*-value = 0.029; Figure 2b). FIGO-based stratification showed a difference in OS (*p*-value = 0.0019; Figure 2c), with 76.3% (95% CI [72.1; 80.5]) and 53.4% (95% CI [45.6; 61.1]) estimated to be alive at 5 years (FIGO stages III and IV, respectively). OS did not differ according to the presence of maintenance treatment, with an estimated 5-year OS of 68.7% (95% CI [63.4; 74.1]) and 70.3% (95% CI [64.8; 75.7]) for presence and absence, respectively (*p*-value = 0.551; Figure 2d).

Concerning mPFS2, a median value of 51.4 months (95% CI [47.6; 67]) was observed. Patients with FIGO stage III and stage IV disease exhibited a mPFS2 of 67.0 (95% CI [50.4; NR]) and 45.8 months (95% CI [38.8; 51.2]), respectively. *BRCA1*- and *BRCA2*-mutated patients had a mPFS2 of 51.2 (95% CI [43.6; 64.2]) and 67.0 months (95% CI [45.8; NR]), respectively. Maintenance treatment during first-line led to an mPFS2 of 51.4 months (95% CI [43.7; NR]), while its absence led to an mPFS2 of 57.7 months (95% CI [45.8; 69.2]).

Median TFST was 31.8 months (95% CI [27.8; 35.1]), with median values of 35.2 (95% CI [28.2; 40.8]) and 27.8 (95% CI [23.8; 31.3]) months for FIGO stage III and stage IV diseases, respectively. Regarding *BRCA* mutation status, *BRCA1*- and *BRCA2*-mutated cases exhibited a mTFST of 29.5 (95% CI [26.2; 34.3]) and 35.5 (95% CI [25.8; 53.4]) months, respectively.

## 4. Discussion

The present analysis of the French national multicentric ESME-OC database allowed for the presentation of real-world data concerning clinical features and survival outcomes among patients with newly diagnosed advanced *BRCA*-mutated HGEOC treated in specialized centers. In our cohort, most patients were diagnosed with FIGO stage III disease. Data from the ESME-OC database rely on a robust process of data collecting process, reflecting the management of patients in expert centers. To our knowledge, this study is the first to bring specific highlights regarding *BRCA*-mutated HGEOC in real-life, with multiple inputs regarding clinicopathological and treatment characteristics. Indeed, contrary to other databases, such as the Surveillance, Epidemiology, and End Results (SEER) database, the ESME database provides substantial information with respect to mutational status, such as clinicopathological data, treatment patterns, and survival trajectory. Furthermore, in spite of international guidelines, treatment strategies vary across countries [33,34]. As such, our study brings a snapshot of clinical practice in French Comprehensive Cancer Centers.

The most common histological subtype was serous carcinoma and *BRCA1* mutation appeared to be more frequent compared to *BRCA2*, as previously reported in the literature [4]. Interestingly, we observed a global trend towards cytoreductive surgery in almost all cases and the use of maintenance treatment with bevacizumab in the vast majority of patients. High rates of complete and partial response (53.3% and 20.4% of the cases, respectively) are in line with the existing literature, showing enhanced sensitivity of *BRCA*-mutated cases to platinum-based regimens [35]. After a median follow up of 51.7 months, we observed an mPFS of 28.6 months (95% CI [26.5; 32.7]) and an estimated 5-year mOS of 69.2% (95% CI [61.6; 70.3]). Notably, *BRCA1*- and *BRCA2*-mutated cases exhibited a trend towards statistically different mPFS of 28.0 (95% CI [24.4; 32.3]) and 33.3 months (95% CI [26.7; 46.1]), respectively (*p*-value = 0.0537). Regarding overall survival, a statistical difference was observed between *BRCA1*- and *BRCA2*-mutated patients, with 64.5% (95% CI [59.7; 69.2]) and 82.5% (95% CI [76.6; 88.5]), respectively (*p*-value = 0.029). Presence and absence of maintenance treatment led to an mPFS of 28.5 (95% CI [25.4; 33.3]) and 29.5 (95% CI [23.1; 33.6]) months, respectively, revealing an absence of difference (*p*-value = 0.5710). Although bevacizumab previously gave conflicting results (notably regarding OS), the present ones should be taken with caution, owing to the descriptive rationale of our study. Indeed, we observed that in patients with a FIGO stage IV disease, maintenance treatment was provided in 36.1% of the cases, while it was not in 29.4% of the cases. These data may have led to a confounding effect, with more tendency to prescribe bevacizumab with more extended disease. Furthermore, rates of cytoreductive surgery (i.e., presence versus absence of residual disease) were not reported in the ESME-OC, limiting the interpretation of physician decision-making regarding prescription of bevacizumab.

Nevertheless, our data were consistent with the literature. Indeed, the randomized clinical trial ICON-7 (NCT00483782), which evaluated the impact of bevacizumab maintenance, reported an mPFS of 19.9 (95% CI [19.1; 22.0]) months in patients treated with platinum-based chemotherapy plus bevacizumab maintenance therapy, versus 17.5 (95% CI [15.7; 18.7]) months with platinum-based chemotherapy only (hazard ratio for progression or death (HR) 0.93; 95% CI 0.83–1.05; *p*-value = 0.25) [36]. Notably, this clinical trial included all-stage ovarian cancers. The GOG-0218 study (NCT00262847) aimed to assess the addition of bevacizumab in de novo advanced stage III (incompletely resectable) or stage IV epithelial ovarian cancers [37]. The mPFS reported was 10.3 months in the control group (chemotherapy only) versus 14.1 months in the bevacizumab group (HR 0.717; 95% CI 0.625–0.824; *p*-value < 0.001). Furthermore, the PAOLA-1 (NCT02477644) randomized clinical trial, which compared bevacizumab versus bevacizumab plus olaparib as first-line maintenance treatment in patients with EOC, reported 21.7 months in patients with bevacizumab [18].

Regarding observational data in real-world settings, the recently published EpOCa Greek study, which included 154 patients, of whom 19 were *BRCA*-mutated, reported an mPFS of 22.5 months (95% CI [19.8–29.2]) when using chemotherapy plus bevacizumab [38]. The GINECO ENCOURAGE cohort, which included 468 French patients (of whom the vast majority had an unknown *BRCA* status), reported an mPFS of 17.4 (95% CI, 16.4–19.1) months with maintenance therapy [39]. Regarding *BRCA*-mutated patients with HGEOC, an mPFS of 29 months was reported through the analysis of 331 patients treated in 15 MITO centers in Italy. Nevertheless, it included all FIGO stages and the inclusion period was 1995–2017 [9]. Notably, our data regarding improved survival in the context of *BRCA2* mutations reinforce previous observations [10,34,39,40].

Our study has some limitations. Although it is based on prospectively collected data, it is based on a retrospective design, with possible selection and treatment biases. The screening process employed for selection of the patients potentially led to selection bias.

Nevertheless, regarding treatment, the short period of inclusion (2011–2016) led to substantially lower risk of such bias. Indeed, guideline-orientated treatments in reference cancer centers leads to quite homogeneous treatment patterns. Although the ESME-OC reflects practices in expert centers, it does not reveal the entire diversity of *BRCA*-mutated HGEOC management in France. As for any data collection, misclassification bias could have emerged. Another potent limitation is the absence of distinction between germline and somatic *BRCA* mutations. Finally, few patients in our study received maintenance with PARPi, although it recently emerged as the new standard of care for advanced HGEOC, notably in the context of *BRCA*-mutated cases, either as monotherapy (olaparib or niraparib) or as combination (olaparib plus bevacizumab) [40,41,42]. Nevertheless, our data provide worthwhile information regarding the treatment and outcomes patterns in real clinical practice regarding a *BRCA*-mutated population. As such, this will allow for evaluation of the impact of adjunction of PARPi in the therapeutic arsenal with subsequent analyses of real-world data.

## 5. Conclusions

This ESME-OC database-derived analysis provides real-world data concerning patients with de novo *BRCA*-mutated advanced HGEOC. It provides researchers with extensive description of clinicopathological, management, and differential survival data regarding this specific population, prior to the advent of the PARPi era. Future real-world data including these molecules during a patient’s journey will enable monitoring of their effect in clinical routine.

## Figures and Tables

**Table 1:** Baseline characteristics.

		*BRCA1* (N = 191)	*BRCA2* (N = 75)	Total (N = 266)
***BRCA* Mutation Type**	N			266
	*BRCA1* only			187 (70.3%)
	*BRCA2* only			75 (28.2%)
	*BRCA1* vs. *BRCA2* unspecified			2 (0.8%)
	Both *BRCA1* and *BRCA2*			2 (0.8%)
**Age**	N	191	75	266
	Median (min–max)	54.5 (33–80)	62.6 (45–81)	56.8 (33–81)
**Performance Status**	N (missing cases)	49 (142)	30 (45)	79 (187)
	0	17 (34.7%)	15 (50.0%)	32 (40.5%)
	1	24 (49.0%)	13 (43.3%)	37 (46.8%)
	2	5 (10.2%)	2 (6.7%)	7 (8.9%)
	3	3 (6.1%)	0	3 (3.8%)
			0	0
**FIGO Stage**	N	191	75	266
	III	129 (67.5%)	49 (65.3%)	178 (66.9%)
	IV	62 (32.5%)	26 (34.7%)	88 (33.1%)
**Histological Type**	N	191	75	266
	Serous and endometrioid	5 (2.6%)	3 (4.0%)	8 (3.0%)
	Serous only	185 (96.9%)	72 (96.0%)	257 (96.6%)
	Endometrioid only	1 (0.5%)	0 (0.0%)	1 (0.4%)
	Serous or endometrioid	0 (0.0%)	0 (0.0%)	0 (0.0%)
**Invasion Sites of Ovarian**	N (missing casess)	190 (1)	74 (1)	264 (2)
**Cancer at Diagnosis**	Ovaries	140 (73.7%)	42 (56.8%)	182 (68.9%)
	Uterin tubes	88 (46.3%)	25 (33.8%)	113 (42.8%)
	Peritoneum	143 (75.3%)	57 (77.0%)	200 (75.8%)
	Nodes	65 (34.2%)	21 (28.4%)	86 (32.6%)
	Colon/small intestine	46 (24.2%)	15 (20.3%)	61 (23.1%)
	Other	158 (83.2%)	62 (83.8%)	220 (83.3%)
**Other Cancer History**	N (missing cases)	190 (1)	74 (1)	264 (2)
	No	188 (98.9%)	73 (98.6%)	261 (98.9%)
	Yes	2 (1.1%)	1 (1.4%)	3 (1.1%)
**If Yes, Type of Cancer**	Colorectal	1 (50.0%)	0 (0.0%)	1 (33.3%)
	Breast	1 (50.0%)	1 (100.0%)	2 (66.7%)
**Family History of**	No	75 (39.3%)	41 (54.7%)	116 (43.6%)
**Breast/Ovarian Cancer**	Yes	95 (49.7%)	24 (32.0%)	119 (44.7%)
	Not available	21 (11.0%)	10 (13.3%)	31 (11.7%)

*BRCA1* actually encompasses patients with either a *BRCA1* mutation (*n* = 187) or a dual *BRCA1/BRCA2* mutation (*n* = 2) or the absence of gene affected (*n* = 2).

**Table 2:** Treatment characteristics.

		*BRCA1* (N = 191)	*BRCA2* (N = 75)	Total (N = 266)
**Treatment Lines (Number)**	N (missing cases)	191 (0)	75 (0)	266 (0)
	1 line	68 (35.6%)	31 (41.3%)	99 (37.2%)
	2 lines	44 (23.0%)	23 (30.7%)	67 (25.2%)
	3 lines	34 (17.8%)	8 (10.7%)	42 (15.8%)
	At least 4 lines	45 (23.6%)	13 (17.3%)	58 (21.8%)
**≥1 Surgery with Resection**	N (missing cases)	191 (0)	75 (0)	266 (0)
	No	8 (4.2%)	4 (5.3%)	12 (4.5%)
	Yes	183 (95.8%)	71 (94.7%)	254 (95.5%)
**Time of 1st Resection**	N (missing cases)	183 (0)	71 (0)	254 (0)
	Before first-line	12 (6.6%)	1 (1.4%)	13 (5.1%)
	Start of first-line	87 (47.5%)	27 (38.0%)	114 (44.9%)
	During first-line	81 (44.3%)	41 (57.7%)	122 (48.0%)
	After the end of first-line	3 (1.6%)	2 (2.8%)	5 (2.0%)
**Best Response in the 1st Line**	N (missing cases)	114 (77)	38 (37)	152 (114)
	Complete	60 (52.6%)	21 (55.3%)	81 (53.3%)
	Partial	21 (18.4%)	10 (26.3%)	31 (20.4%)
	Stable	11 (9.6%)	1 (2.6%)	12 (7.9%)
	Progression	7 (6.1%)	3 (7.9%)	10 (6.6%)
	Other	15 (13.2%)	3 (7.9%)	18 (11.8%)
**Best Response in the 2nd Line**	N (missing cases)	75 (66)	29 (26)	104 (92)
	Complete	22 (29.3%)	11 (37.9%)	33 (31.7%)
	Partial	16 (21.3%)	4 (13.8%)	20 (19.2%)
	Stable	14 (18.7%)	4 (13.8%)	18 (17.3%)
	Progression	11 (14.7%)	4 (13.8%)	15 (14.4%)
	Other	12 (16.0%)	6 (20.7%)	18 (17.3%)
**Maintenance Treatment**	N (missing cases)	191 (0)	75 (0)	266 (0)
	No	83 (43.5%)	36 (48.0%)	119 (44.7%)
	Yes	108 (56.5%)	39 (52.0%)	147 (55.3%)
**Type of Maintenance**	N (missing casess)	108 (0)	39 (0)	147 (0)
	Bevacizumab only	86 (79.6%)	30 (76.9%)	116 (78.9%)
	Bevacizumab + other *	16 (14.8%)	3 (7.7%)	19 (12.9%)
	Other	6 (5.6%)	6 (15.4%)	12 (8.2%)
**Number of Cycles of Platinum-Based** **Chemotherapy**	N	186 (5)	73 (2)	259 (7)
	Mean ± SD	6 ± 3	6 ± 2	6 ± 2
	Median	6	6	6
	Min; Max	1; 22	2; 15	1; 22

*BRCA1* actually encompasses patients with either a *BRCA1* mutation (*n* = 187) or a dual *BRCA1/BRCA2* mutation (*n* = 2) or the absence of gene affected (*n* = 2). * “other” refers to patients included in clinical trials, with complementary treatments added to bevacizumab.

## Data Availability

The datasets analyzed during the current study are available in the ESME-OC database. The database of the ESME program or the database of the OC cohorts are currently not accessible. For any specific demand, please contact the corresponding author. Each demand will be examined on a case-by-case basis by the scientific committee.
